# *In situ* wet-cell TEM observation of gold nanoparticle motion in an aqueous solution

**DOI:** 10.1186/1556-276X-7-598

**Published:** 2012-10-29

**Authors:** Xin Chen, Jianguo Wen

**Affiliations:** 1 Key Laboratory for Ultrafine Materials of Ministry of Education, School of Materials Science and Engineering, East China University of Science and Technology, Shanghai, 200237, People's Republic of China; 2 Shanghai Key Laboratory of Advanced Polymeric Materials, School of Materials Science and Engineering, East China University of Science and Technology, Shanghai, 200237, People's Republic of China; 3 Department of Materials Science and Engineering, University of Illinois at Urbana-Champaign, Urbana, IL, 61801, USA; 4 Electron Microscopy Center and Materials Science Division, Argonne National Laboratory, Argonne, IL, 60439, USA

**Keywords:** *In situ* transmission electron microscopy, Gold, Nanoparticles, Wet cell

## Abstract

*In situ* wet-cell transmission electron microscopy (TEM) technology enables direct observation of nanomaterials in a fully hydrated environment with high spatial and temporal resolution, which can be used to address a wide range of scientific problems. In this paper, the motions of approximately 5-nm sized gold nanoparticles in an aqueous solution are studied using the wet-cell TEM technology. It is observed that gold nanoparticles can be either in a single particle or cluster forms, and dynamic displacement and rotation motions are observed for both forms in the solution. Under electron beam irradiation, nanoparticles in some clusters gradually fused together; sometimes they also showed dramatic growth behavior. Mechanisms for the motion and growth of the particles/clusters are discussed.

## Background

Nanoparticle assemblies are often achieved involving liquids. Real-time observation of nanoparticle assembly and dynamics is thus of great importance [1,2]. *In situ* transmission electron microscopy (TEM) techniques provide a local probe of structure and dynamics that other techniques cannot observe readily. *In situ* observation provides dynamic information about nanosystems, which is difficult to obtain by other techniques. Conventional TEM requires drying of samples in order to be compatible with vacuum. The structural features of the sample can change significantly during the process. Thus, for samples prepared in liquids, it would be ideal if it can be observed directly with TEM. With the development of robust silicon nitride (Si_3_N_4_) membrane windows for the *in situ* cell [3], the construction of wet-cell and *in situ* observation of liquids becomes readily possible inside TEM. Applications using the wet-cell technology are upsurging, and further exciting development is expected in the future [1]. For examples, this technique has been applied to the observation of electrochemical dynamic procedure of Cu [3] and Ni [4] nanoclusters, electron beam-induced growth of Pt [5] and lead sulfide nanocrystals [6] in liquid, semiconductor nanorod embedded in liquid crystal cells for optoelectronic applications [7], Al_2_O_3_ nanoparticles and carbon nanotubes in water [8], and biological cells [9]. Earlier liquid cell TEM reactor yielded a spatial resolution of only 5nm, but recent development has improved the resolution to the sub-nanometer range [5]. The development in Berkeley using graphene sheet to replace Si_3_N_4_ even pushed the wet-cell TEM imaging resolution to the atomic level [10].

Zheng et al. recently made an analysis on gold nanoparticle diffusion during liquid evaporation [11]. In addition, Grogan and Bau reported observation with *in situ* STEM on gold clusters in an aqueous solution using a lower electron beam energy of 20 keV to test the liquid cell hermeticity. However, the image resolution is poorer due to the lower electron energy [12].

High spatial resolution TEM requires both high voltage and relatively high electron beam flux. Local heating and structural transformations may occur during observation due to the electron beam irradiation [13]. Previous reports have shown that electron beam can initiate nanoparticle nucleation and growth in a liquid [5]. Such beam effect needs to be carefully addressed in wet-cell TEM experiments.

In this paper, we report an *in situ* observation of gold nanoparticles in aqueous water solution using the wet-cell TEM technology. Sub-nanometer resolution images were obtained. Dynamic motion and dramatic growth of clusters of gold nanoparticles have been observed. These observations allow a discussion of electron beam effect on the growth of nanoparticle clusters.

## Methods

**Figure 1:**
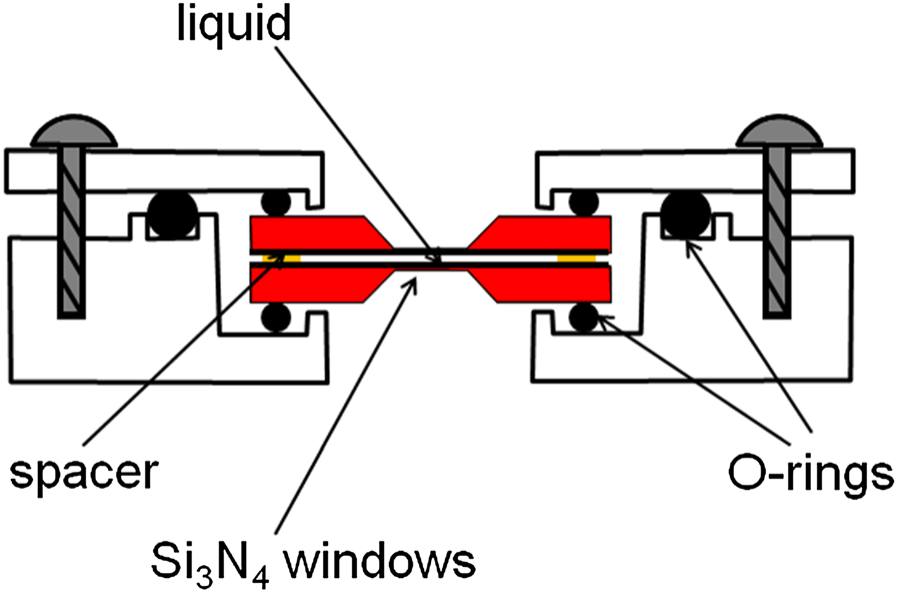
**A schematic of the wet**-**cell for *in situ *TEM analysis.**

Gold nanoparticles are studied with an O-ring sealed clamp on wet-cell developed earlier at the University of Illinois at Urbana-Champaign [8]. As shown in Figure 1, the cell utilizes two Si_3_N_4_ window grids to confine the liquid. The cell seals via three O-rings that couple the grids and the top and bottom pieces of the enclosure together. The design utilizes commercial silicon nitride grids as the substrates and a fixed reusable cell, which is simply assembled with O-rings and screws, limiting the need for complex microfabrication procedures to generate appropriate windows for each experiment. Once the grids are put in place and the liquid is loaded, the cell can be completely assembled within few minutes. Gold nanoparticles (5 nm in diameter) dispersed in DI water (Nanocs Inc., New York, USA, GNP0001-5 (20 ml 0.01% Au)) were tested *in situ* in TEM chamber. Si_3_N_4_ window grids (50-nm thick) from Ted Pella, Inc. (CA, USA) were used to sandwich the liquid in between. A JEOL 2010 LaB_6_ TEM system (JEOL Ltd., Tokyo, Japan) was used for the observation, which operated with a 200-kV electron acceleration voltage.

## Results and discussion

**Figure 2:**
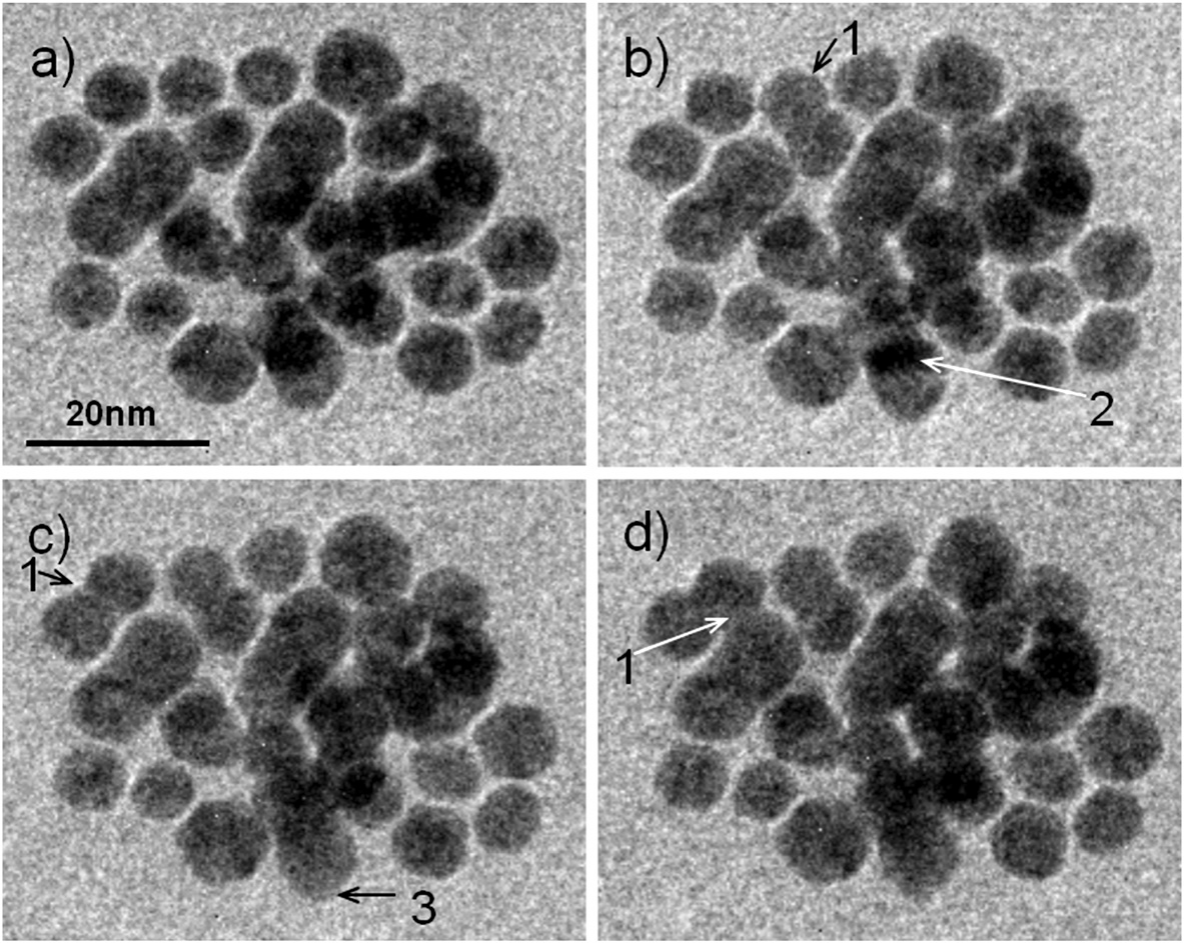
**Wet**-**cell TEM observation of gold nanoparticle motion in water.** (**a**) 0, (**b**) 2, (**c**) 3, and (**d**) 4 min. Arrow 1, particles move together and merge; 2, particles move apart so the overlapped dark area becomes smaller; 3, the particle bottom become rounder, suggesting that the particle might be rotating.

Figure 2 shows a cluster of gold nanoparticles observed in an aqueous solution sealed with the wet-cell. The roughened particle shapes indicate that they are faceted nanocrystals. Diffraction contrast fringes can be seen in individual particles, confirming the crystalline structure of the particles. The spatial resolution of the images is about 0.5 nm. Panels a, b, c, and d of Figure 2 were obtained at 0, 2, 3, and 4 min, respectively. The particles are located at relatively fixed locations with time, without showing Brownian motion, suggesting they might be attached on the silicon nitride window without being able to freely move in the liquid. Changes in the particle morphology occurred over time. In the figure, arrow 1 points to locations where particles coalesce with time. Arrow 2 points to an overlap region with darker contrast due to 3D arrangement of the nanoparticles. From Figure 2a,b,c, we see that overlapped region is reducing in size with time, suggesting that the two particles are in different planes and are moving apart from each other. Arrow 3 points to the bottom contour of the moving down particle. From Figure 2a,b,c, we see that the contour is changing shape with time from a flat bottom line to a relatively rounder one, suggesting the particle was rotating as it moves down.

**Figure 3:**
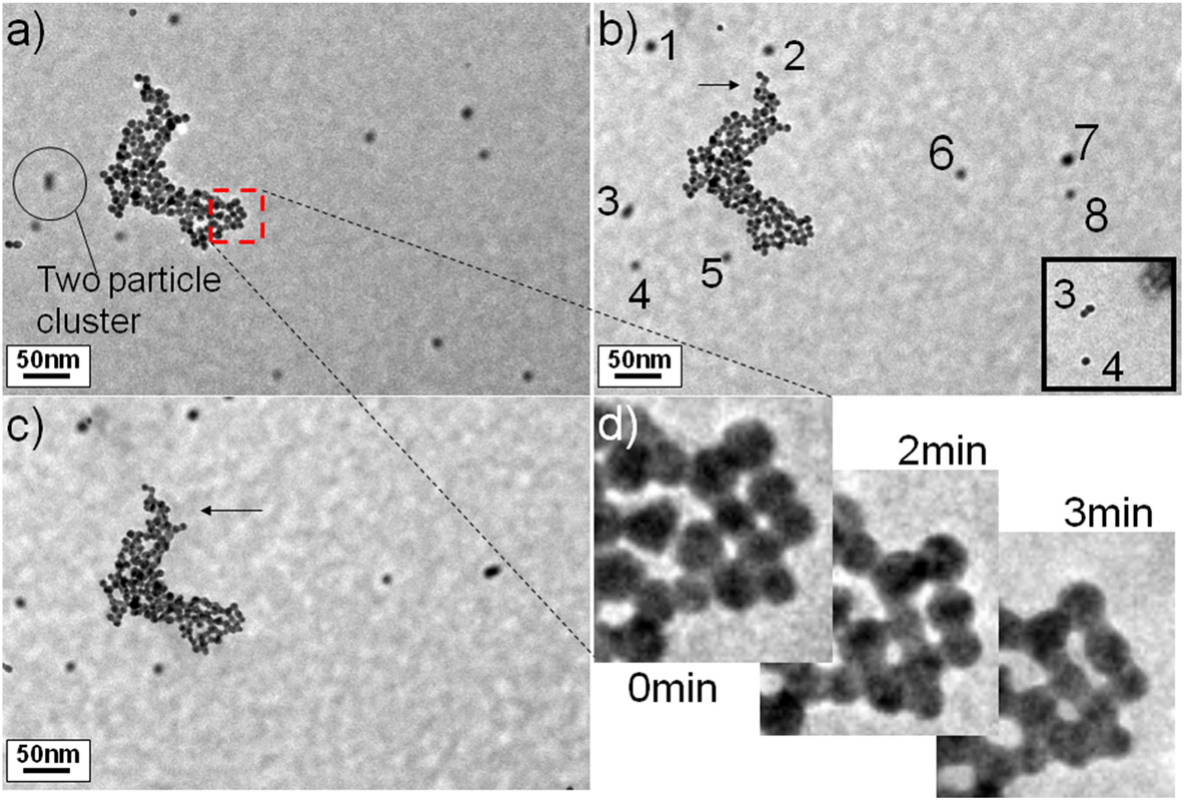
**A gold cluster rotating in liquid**, **with particles roaming randomly in the background.** (**a**) 0, (**b**) 2, and (**c**) 3 min;(**d**) Magnification of partial of the cluster from (**a**), (**b**), and (**c**). Inset of (**b**) is an image focusing on the background particles, showing 3 is a two particle cluster.

The above changes in the gold particles do not have to happen in a liquid. When we put gold nanoparticles on a dry Si_3_N_4_ grid, similar behavior was observed. It has also been reported earlier in TEM analysis that for nanoparticles, coalescing, attachment, rotation, and agglomeration on a dry substrate can happen at temperatures much lower than the melting temperature of a bulk material [14]. For verification of the particle dynamics in solution, further observations were made. As shown in Figure 3, constant motions of gold particles and clusters in the fluid were seen. Panels a, b, and c of Figure 3 were taken at 0, 2, and 3 min, respectively. We see that the gold cluster in the middle changes angle with time, and there are individual dots that are moving constantly. The center cluster and the individual dots are not in the same depth in the cell. The cluster has a point fixed on the grid and thus can only rotate with time, but not move in location. By adjusting the focus of the TEM, we found that the individual dots are at a different focus depth and thus should be deeper in the liquid. Eight dots are labeled out which we used to track the motion with time. From the figure, we see the dots are moving around while changing relative positions to each other with time. Dots 3 and 4 are moving apart with time, while dots 7 and 8 moved together. Dot 3 is changing shape because it is a two-particle cluster (see inset of Figure 3b) which is rotating while moving in the liquid. The actual motions of the particles and clusters in the wet-cell are more dramatic than we directly saw in the figure. The big cluster was observed to frequently change angle with time and rotate from one end to the other (approximately 30°) within a fraction of a second; there are also particles and clusters that move much faster than the eight labeled dots. The fact that the big cluster can rotate quickly suggests that the individual dots could also move much quicker if freely suspend in the liquid. The moderate displacement observed on these dots suggests that they might have been slowed down due to the interaction from the silicon nitride window. Actually, when we adjust focus on these dots, we found that they are on about the same focus plane, supporting that they should be close to a window plane instead of freely suspended in different depths in the liquid. It has been estimated by White et al. [15] that, for Brownian motion of 4-nm diameter particles in water, a mean square displacement <*x*^2^>^1/2^ of approximately 10 μm is expected within a 1/3s drift time, which is much larger than a regular TEM imaging width. Thus, the fact that the particles remain visible for more than one frame during the TEM observation indicates that the Brownian motion is greatly suppressed by the interaction such as that from the window. The observation shown in Figure 2 can be viewed as an extreme case. When the particles are tightly attached to a window, their motion in the liquid almost stopped.

Figure 3d is the enlargement of one section of the center cluster, in which we see individual gold nanoparticles in the cluster are merging with time, confirming that the particle agglomeration behavior is happening in the liquid environment. The cluster looks like individual beads that are attached to each other in the beginning but became more like a web made up of nanowires after 3 min of electron beam irradiation. It is significant that no bubbling in the liquid is seen during the observation, which indicates that the welding procedure is happening even below the boiling temperature of water. It has been calculated that the temperature change in a wet-cell under electron beam irradiation is generally much smaller than 1 K [15]. Dramatic changes in gold nanoparticles such as dissolution had been previously observed under relatively high electron doses of 8 × 10^5^ e/nm^2^[16]. In our experiment, with the longer observation time of several minutes, the electron dose may accumulate to beyond a limit that affects the observable gold crystal morphology.

**Figure 4:**
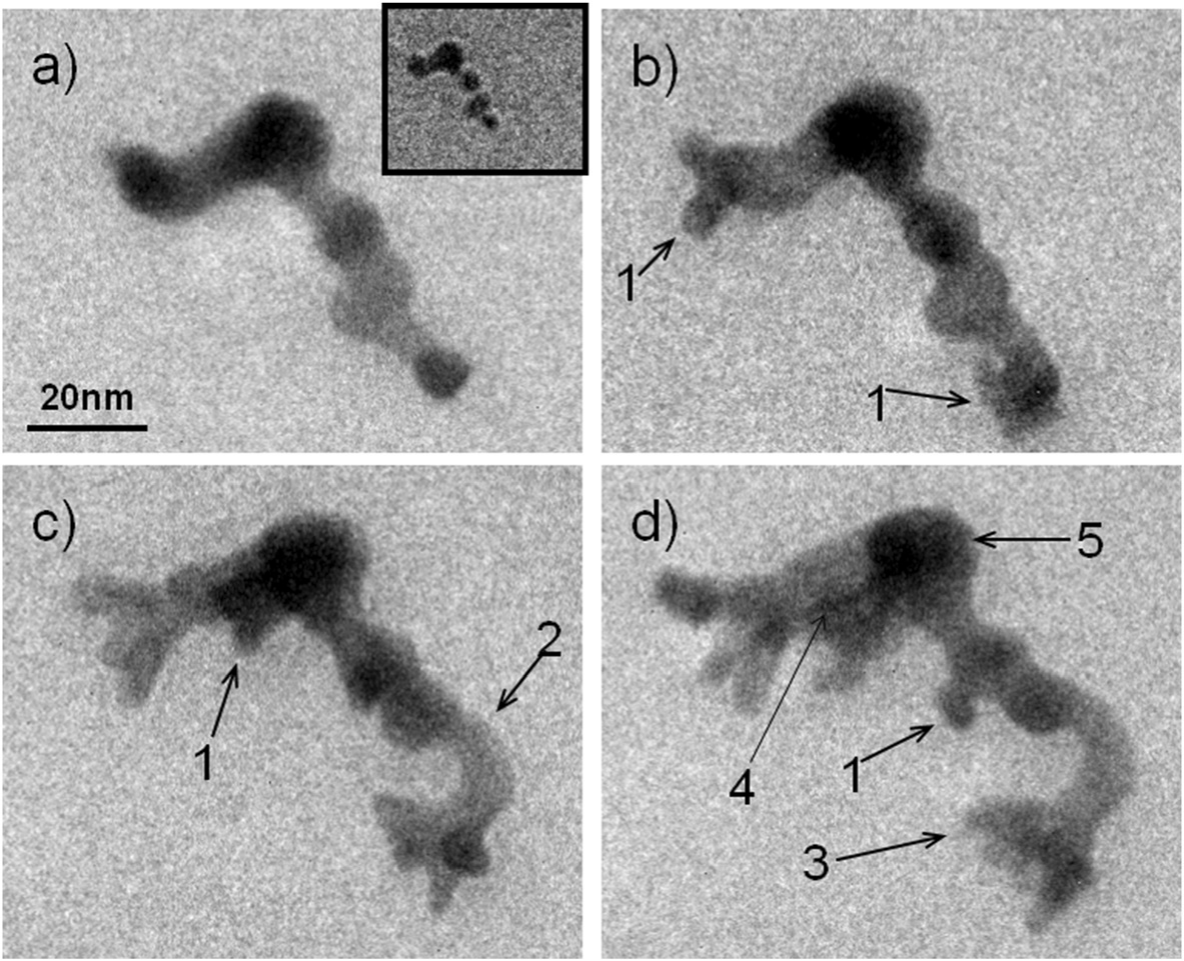
**A nanocluster that grows dramatically in size with time (a) 0, (b) 3, (c) 8, and (d) 12 min: Arrow 1, new branches grow out; 2, nanowire in the middle of the cluster bends up and grows longer; 3, a branch on the nanocluster became less clear due to motion in the water or drifting into different depth of the liquid.** Inset of (**a**) is the nanocluster micrograph that was taken before image adjustment.

A more dramatic shape change in gold nanoparticle cluster is recorded as shown in Figure 4. The inset of Figure 4a shows a six-particle cluster during image adjustment. A bubble has been seen nearby, confirming the cluster is submerged in the liquid. Similar to Figure 2, this cluster is relatively fixed to a location, without making quick rotations or floating around to large distances. However, in Figure 4a, we see that after the image adjustment, the particles not only fully merged together, but also changed shape significantly, with some short nanowires growing up in between. Figure 4b,c,d shows that as time passes (3, 8, and 12 min), the cluster continues to grow up. Arrow 1 points to locations where new branches grow out from the cluster. Arrow 2 points to where the nanowire bends during growth. Arrow 3 points to the tip of the cluster which becomes blurred with time; this could be due to the bending of the nanocluster into the liquid, thus moving out of the focus of the microscope.

Although a longer beam exposure time (12 min in Figure 4 vs. 3 to 4 min in Figures 2 and 3) can be used to partially explain such a dramatic shape change, it is unusual to see that the cluster increases in size greatly with time. Chemical reactions could have occurred that transported materials into the cluster from regions away from the electron beam. Although a noble metal, gold can form many diverse compounds. One possibility is that gold reacted with water under the high energy electron bombardment and formed gold oxide or gold hydroxide; however, this growth mechanism is not fully sustainable. After all gold atoms in the cluster are oxidized, the volume increase will stop. As the cluster in Figure 4 is fixed in location without being able to move freely around in the liquid, it must be closely attached to the silicon nitride window, and wetting of the gold on the window might also account for the morphology change. Arrow 4 in Figure 4d points to a black line in the growing cluster, suggesting a trace for material migration during the growth. Compared with Figure 4c, it appears that the round dark region near the top center (region 5 in Figure 4d) is the source region for the mass redistribution, and the growing branches to the left are the regions where the materials are transported toward. The nanowires are in much lighter color in these TEM bright field images than the earlier gold particles, suggesting that they are much thinner, thus results in a relatively large area increase. This supports the idea that the gold wets the window under electron beam irradiation. In comparison, we irradiated gold nanoclusters on a dry grid for the same amount of time. Besides the simpler merging behavior like in Figure 2, no such dramatic growth behavior was observed, suggesting that water might have played a role in helping catalyze the nanowire growth. Recently, Zheng et al. reported *in situ* TEM observation of Pt_3_Fe nanorod growth in solution [17], in which Pt_3_Fe nanoparticles attach and coalesce into nanoparticle chains. The chains were winding and markedly flexible, and gradually turned into nanowires through mass redistribution procedure. Most of the nanowires remain polystalline and twisted for an extended period of time. Yuk et al. [10] further reported *in situ* TEM observation of Pt nanoparticles coalescing in liquid. These observations are similar to our results here. It is not fully excluded that there is the possibility that gold dissolution happened somewhere in the liquid, which got redeposited onto the growing cluster under the beam interaction. Further study on the gold morphology change under electron beam irradiation in solution is still needed.

## Conclusions

In summary, we report the observation of gold nanoparticles in water solution using *in situ* wet-cell TEM technology. The gold nanoparticle system showed a variety of dynamic behaviors in the aqueous solution involving single particles, particle clusters, and nanowires, which include dynamic displacement and rotation motions, fusion of particles, and even dramatic size growth behavior under the electron beam. The fusion and dramatic growth of the particles happened at temperatures much lower than the gold melting temperature. Random motions of the gold particles and clusters are greatly suppressed by the drag from the silicon nitride windows.

## Competing interests

The authors declare that they have no competing interests.

## Authors' contributions

XC carried out the *in situ* TEM studies, participated in the wet-cell TEM technique advancement, and drafted the manuscript. JW made substantial contribution to TEM wet-cell development and participated in the TEM study. All authors read and approved the final manuscript.

## Authors' information

XC is currently a professor at the School of Materials Science and Engineering, East China University of Science and Technology, Shanghai, China. JW is a materials scientist at Electron Microscopy Center and Materials Science Division, Argonne National Laboratory, Argonne, IL, USA
